# First evidence of marine turtle gastroliths in a fossil specimen: Paleobiological implications in comparison to modern analogues

**DOI:** 10.1371/journal.pone.0302889

**Published:** 2024-05-06

**Authors:** Giovanni Serafini, Caleb M. Gordon, Jacopo Amalfitano, Oliver Wings, Nicole Esteban, Holly Stokes, Luca Giusberti

**Affiliations:** 1 Dipartimento di Scienze Chimiche e Geologiche, Università di Modena e Reggio Emilia, Modena, Emilia-Romagna, Italy; 2 Department of Earth & Planetary Sciences, Yale University, New Haven, CT, United States of America; 3 Centro di Ateneo per i Musei, Università Degli Studi di Padova, Padova, Veneto, Italy; 4 Naturkundemuseum Bamberg, Bamberg, Bavaria, Germany; 5 Staatliche Naturwissenschaftliche Sammlungen Bayerns, München, Germany; 6 Faculty of Science and Engineering, Swansea University, Swansea, Wales, United Kingdom; 7 Dipartimento di Geoscienze, Università Degli Studi di Padova, Padova, Veneto, Italy; University of Silesia, POLAND

## Abstract

Semi-articulated remains of a large chelonioid turtle from the Turonian strata (Upper Cretaceous; ca. 93.9–89.8 Myr) near Sant’Anna d’Alfaedo (Verona province, northeastern Italy) are described for the first time. Together with the skeletal elements, the specimen also preserves pebbles inside the thoracic area which are lithologically distinct from the surrounding matrix. These allochthonous clasts are here interpreted as geo-gastroliths, in-life ingested stones that resided in the digestive tract of the animal. This interpretation marks the first reported evidence of geophagy in a fossil marine turtle. SEM-EDS analysis, together with macroscopic petrological characterization, confirm the presence of both siliceous and carbonatic pebbles. These putative geo-gastroliths have morphometries and size ranges more similar to those of gastroliths in different taxa (fossils and extant) than allochthonous “dropstone” clasts from the same deposit that were carried by floating vegetation A dense pitted pattern of superficial erosion is microscopically recognizable on the carbonatic gastroliths, consistent with surface etching due to gastric acids. The occurrence of a similar pattern was demonstrated by the experimental etching of carbonatic pebbles with synthetic gastric juice. Gut contents of modern green sea turtles (*Chelonia mydas*) were surveyed for substrate ingestion, providing direct evidence of geophagic behavior in extant chelonioids. Comparison with modern turtle dietary habits may suggests that the pebbles were ingested as a way to supplement calcium after or in preparation for egg deposition, implying that the studied specimen was possibly a gravid female.

## Introduction

Gastroliths are any hard objects without a caloric value that are found in or have passed through the digestive tract of an animal [1–3]. Following the classification of Wings [1], gastroliths can be subdivided into *bio-gastroliths*, mineral concretions physiologically formed in the guts of decapod crustaceans prior to ecdysis [4–6]; *patho-gastroliths*, stone-like pathological growths nucleated around ingested materials (e.g. hairs or vegetal fibers by grazing mammals; [7]; i.e. bezoars); and *geo-gastroliths*, sedimentary particles deliberately or accidentally ingested by animals [2,8]. Geo-gastroliths are the direct result of geophagy, the ingestion of rocks or soil by an animal. This behavior is widespread in metazoans, particularly in vertebrates [8], and many hypotheses to account for geophagy have been proposed. Common (and not mutually exclusive) explanations of geophagy in both extant and fossil vertebrates fall under the following categories:

- *Alimentary hypotheses* posit that sediment is ingested as mineral supplement [9,10], for trituration of ingesta [2,11,12], for mixing of ingesta [13] or for stomach cleansing [14,15].
- *Buoyancy control hypotheses* posit that ingested stones function as ballast and hydrostatic control in aquatic vertebrates [1,8,16] and reference therein).
- *Pathological hypotheses* posit *the* deliberate ingestion of sediment to attempt the removal of parasites from stomach/intestine walls [17] or unwilling stress/pathology-induced stone ingestion [18].
- *Accidental ingestion hypotheses* posit that stones have been mistaken as prey items [19], ingested directly with food as by-catch [20], or ingested indirectly by the consumption of prey items that themselves contain gastroliths [6,21].

Geo-gastroliths have been reported in various vertebrate clades [1]. Birds are by far the most common reported example of geophagic vertebrates, especially herbivorous taxa with thick-walled gastric mills specialized for grinding hard food [11,13,22], but the phenomenon is also well documented in crocodylians [23,24], various other non-avian sauropsids [8,20,25] and marine mammals [1,8,26]. The fossil record is fairly extensive for gastroliths in vertebrates, since pebbles are easily preserved and contained by the ribcage of the animals during taphonomic processes [13]. Among fossil vertebrates, marine amniotes are well known to host geo-gastroliths [1,8,27], especially sauropterygians, which are regularly found to contain stomach stones [27–30].

However, among fossil pelagic reptiles, marine turtles have never been described with associated gastroliths [8,16]. Taylor [8], while listing occurrences of gastroliths in tetrapods, states that “gastroliths are rare or unknown from marine turtles”. To our knowledge, the only previous reported specimen of fossil marine turtle with preserved sediment particles in the body cavity is a cheloniid from the Rupelian of southern Germany (Unterfeld) described by Alexander & Frey [31]; these particles are however described as naturally occurring mineral concretions nucleated during carcass decomposition and fossilization, rather than geo-gastroliths [31]. Reports of geophagic behavior in extant chelonians are also scarce: extant tortoises have been observed to ingest sediment particles during ecological and veterinary surveys [10), and previous analyses of gut contents suggest at least some sporadic ingestion of sediments by marine chelonioids (e.g. green sea turtles: [32,33]; leatherbacks with accidentally ingested sand: [34]). However, despite occasional reports of geophagy in extant turtles, the widespread nature of geophagic behavior in various other marine amniotes, and the high preservation potential of gastroliths, no gastroliths have yet been described in fossil marine turtles.

Here we describe a large chelonioid turtle (IGVR 91051) from the Upper Cretaceous strata of Scaglia Rossa near Sant’Anna d’Alfaedo (Verona province, northeastern Italy) that preserves gastroliths between its axial and dermoskeletal elements. The turtle fossil record of the Scaglia Rossa is best known for the holotype of *Protosphargis veronensis*, a large pelagic chelonioid of dubious taxonomic affinities [35–37]. IGVR 91051 is a large, semi-articulated individual discovered in the early 2000’s; the specimen still lacks an official description. Our study summarily reports the anatomy and taxonomy of IGVR-91051 but refrains from attempting to resolve the position of *Protosphargis* or IGVR 91051 within Chelonioidea, a task that requires a separate investigation. Our analysis focuses instead on the preserved geo-gastroliths present in the former digestive tract of the specimen, with a detailed characterization of their morphology, composition, surface etching pattern, and function. We also present new direct evidence of stomach stones within modern sea turtles, with the reappraisal of gastric content in specimens of *Chelonia mydas* from the Republic of Seychelles. For the first time modern sea turtle gastroliths are figured and characterized, and their frequency of occurrence is compared between sexes and breeding vs. non-breeding individuals. Our data suggest a long and under-appreciated history of chelonioid geophagy that extends back to the Cretaceous.

### Geological and paleontological context

**Fig 1:**
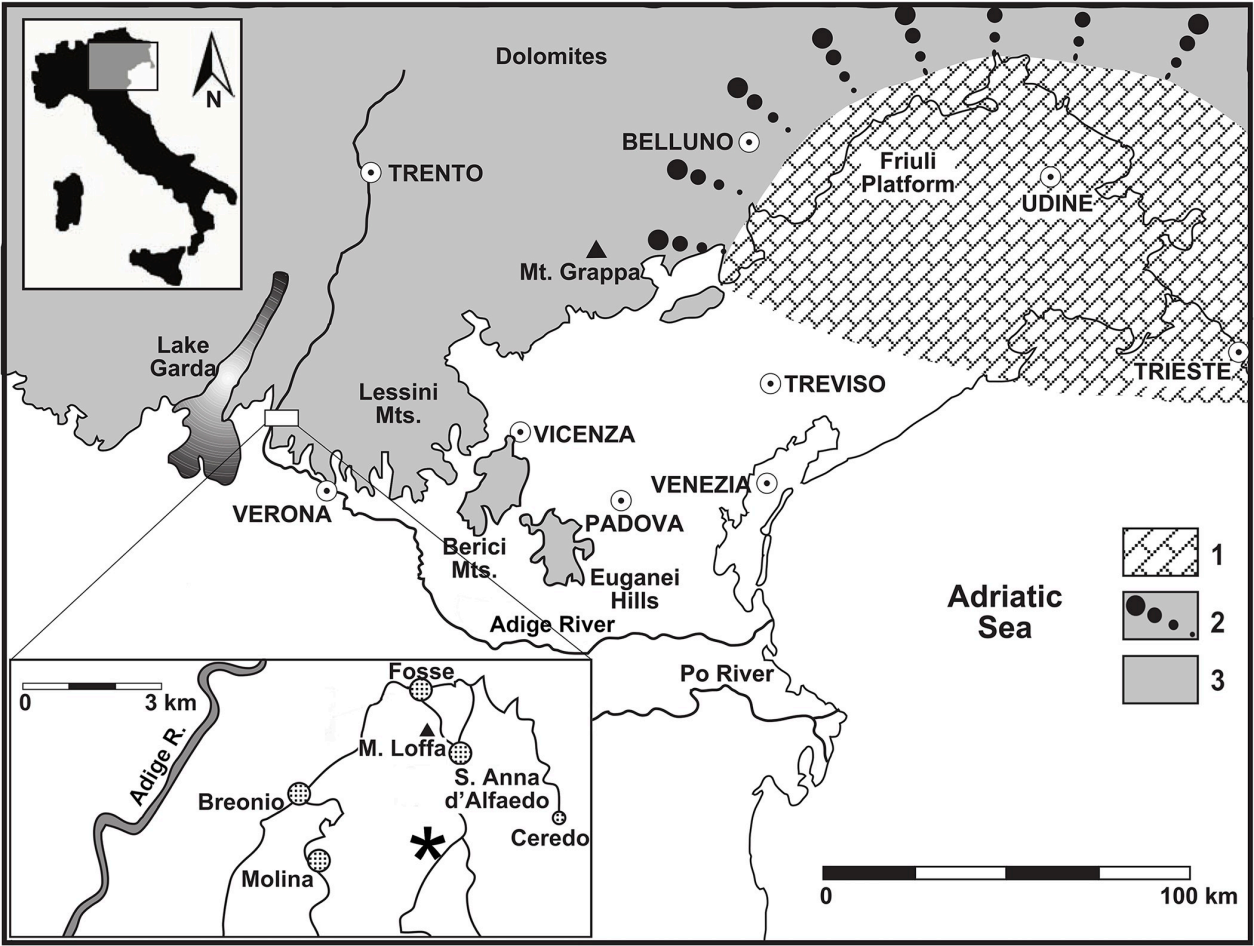
Schematic and simplified paleogeography of the central-eastern Southern Alps during the Late Cretaceous superimposed on present-day geography and indication of the site of finding of IGVR 9105. 1. Shallow-water carbonate platform deposits (Friuli Platform). 2 Slope-resedimented deposits of the Friuli Platform (“Fadalto Limestone”). 3. Basinal pelagic limestones (Scaglia Rossa and equivalent deposits). Modified after [44,57]. Map modified and reprinted from [57] under a CC BY license, with permission from Rivista Italiana di Paleontologia e Stratigrafia, original copyright year 2013.

The specimen was found in a quarry of Scaglia Rossa near the village Sant’Anna d’Alfaedo (Lessini Mountains of Verona Province, northeastern Italy; Fig 1). The Scaglia Rossa (SAA) is a lithostratigraphic unit deposited in the Trento Plateau and Belluno Basin [38,39], in what is now the Veneto region of northeastern Italy, during a time interval that extends from the Turonian to the early Eocene [40,41]. This formation is represented by pink and reddish limestones, cherty limestones and marly limestones rich in planktic foraminifera that were originally deposited in a pelagic/hemipelagic setting [41,42]. Usually, Scaglia Rossa is poor in macrofossils, mainly represented by irregular echinoids and mollusks (ammonites, inoceramids and rudists; [43,44]). Marine vertebrate remains are exceedingly rare (chondrichthyan and teleostean fish, marine turtles and mosasaurs) and mostly come from a peculiar lithofacies known as “lastame” or "Pietra di Prun" (“Prun Stone”) (e.g., [40,42,43,45,46]). The “lastame”, corresponding to the "lithozone 2" of Scaglia Rossa in Lozar & Grosso [39], is a ca. 7-meter-thick package of nodular/subnodular limestone and marly limestone intensively quarried in the Lessini Mountains of Verona province for decorative and building stone (e.g., [47]). “Lastame” dates back to Turonian p.p.-Coniacian p.p. [43,44], but most of the vertebrate skeletons and partial remains so far investigated comes from the middle-upper Turonian interval (e.g., [48]). A sedimentological peculiarity of “lastame” is represented by the presence of allochthonous stones (pebbles and cobbles) in some beds of the lower half of the lithozone (e.g., [43,49]): these exotic clasts, sometimes associated with teredinid tubes, have been interpreted as dropstones, fragments of rocks carried at sea by floating driftwood that released them on the seafloor after decomposition [50,51]. These dropstones are made up of sedimentary, magmatic and metamorphic rocks and their source areas are unknown, although Massari & Savazzi [50] noted some affinities with lithologies of the Upper Cretaceous Lombardian Flysch. Paleodepth interpretation of “lastame” in the Sant’Anna d’Alfaedo area is quite controversial and debated (ranging from 50 to 100 m to full bathyal; [44,52,53]). The vertebrate macrofauna so far recovered in the Lessinian “lastame” includes: the sharks *Cretoxyrhina mantelli*, *Cretodus crassidens*, *Ptychodus* spp., the batomorph *Onchosaurus pharao*, still unidentified teleostean remains, marine turtles (detailed below), and the yaguarasaurinid mosasaurs *Romeosaurus fumanensis* and *R*. *sorbinii* (e.g., [40,42,46,48,54–56]). Vertebrate remains show virtually no current-mediated displacement of bones [40,42,50] and usually represent various degrees of disarticulation of slowly decaying carcasses exposed for extended time intervals on the seabed under low-energy conditions [54]. To our knowledge, none of these fossil vertebrates were recovered from the same beds that yielded the dropstones (see also [50]).

### The “lastame” turtles

**Fig 2:**
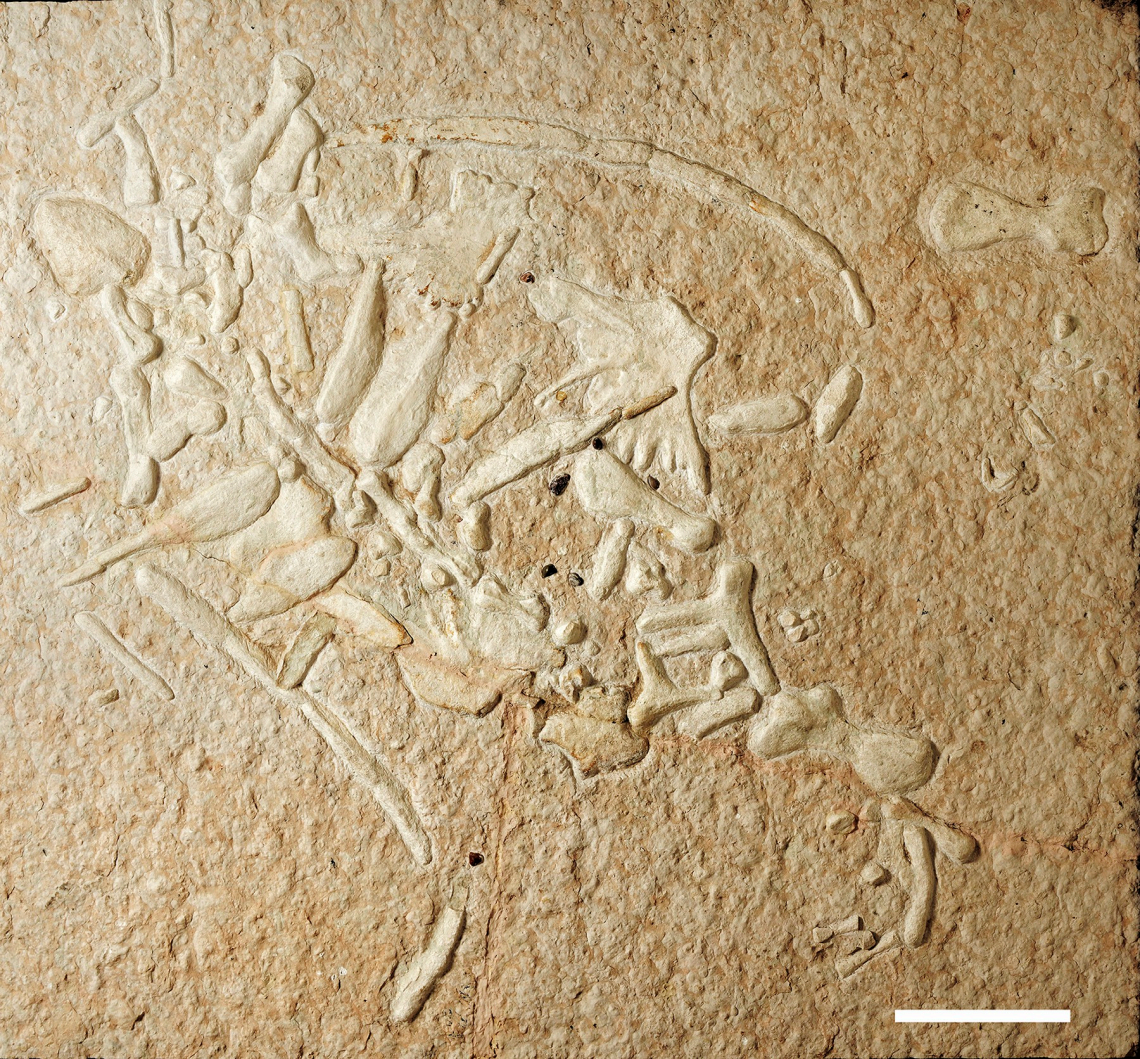
Scaglia Rossa “lastame” slab preserving the protostegid IGVR 9105. Scale bar 30 cm.

Turtle remains from the Lessinian “lastame” are relatively well represented, with a long history of discovery dating back to the mid-19th century. Capellini [35] erected the species *Protosphargis veronensis* based on the first specimen found in 1852 in the surroundings of Sant’Anna d’Alfaedo (in a quarry at Monte Guaiti), which was originally misinterpreted as human remains (e.g., [58–60]); this taxon, after the two exhaustive publications of Capellini [35,61], was only sporadically cited in the literature or in exhibition catalogs, but was never the object of modern studies, despite the finding of new material in the 19th and 20th centuries. Specifically, a large and remarkably complete specimen was found in a quarry at Monte Loffa (Sant’Anna d’Alfaedo, Verona) in 1972 and preliminary ascribed to *P*. *veronensis* (e.g., [43,62]). The fossil, housed at the Museum of Natural History of Verona, was never formally described. The most recent discovery of a possible *Protosphargis* from the study area refers to the semi-articulated specimen herein described (IGVR 91051; Fig 2), recovered in the 2000s in a quarry at Masue, south of S. Anna d’Alfaedo village (Fig 1) and close to the type locality of the holotype of *P*. *veronensis*. A renewed interest in Italian Cretaceous marine turtles is due to De Lapparent [63] and Hirayama [36], who discussed the systematic position of *Protosphargis*. More recently, Chesi & Delfino [37] and Chesi [64] listed and discussed the findings of marine turtles from the Upper Cretaceous of Italy, including *P*. *veronensis* and some undetermined Dermochelyoidae and Cheloniidae.

## Material and methods

IGVR 91051 (Fig 2) is currently housed in the Community Theatre of Sant’ Anna d’Alfaedo (Verona province) on a roughly 170 x 190 cm slab of subnodular pink-reddish marly limestones. The specimen contains 10 allochthonous pebbles (G1 to G10) interpreted as geo-gastroliths.

### Comparative 2D analysis on gastroliths

Pebbles preserved within IGVR 91051 were morphologically characterized following part of the identification route standardized by Wings [1]. To test the hypothesized gastrolith status of these stones, we compared their shape and size to those of abiotically dispersed allochthonous clasts from the same formation, *bona fide* gastroliths recovered from Recent tetrapods, and *bona fide* gastroliths recovered from tetrapod fossils. Following previous researchers [65–70], we measured particle size and shape with three parameters: long axis length (equivalent here to Feret diameter, *sensu* [69]), circularity (4π ∙ stone area/perimeter^2), and roundness (4 ∙ stone area / π ∙ (long axis length)^2). Circularity measures how close in shape a particle is to a circle, whereas roundness measures the angularity or “jaggedness” of its corners [66,68]. Given that these and similar parameters can be measured from two-dimensional projections of a particle on ImageJ [70], we were able to obtain a large dataset of stones by sampling photographs. To measure these parameters, we obtained scaled high-resolution photographs of n = 585 stones from a variety of specimens (S1 Table). These included n = 10 stones preserved within the chelonioid IGVR 91051 (G1–G10), n = 81 allochthonous clasts from Scaglia Rossa “lastame” (on display at the Museo Paleontologico e Preistorico of S. Anna d’Alfaedo, Verona), and n = 494 *bona fide* gastroliths from a combination of extant sea turtles (*Chelonia mydas*), extant crocodilians (*Crocodylus acutus*, *C*. *niloticus*, *C*. *novaguineae*, *Tomistoma schlegelli*), and several extinct aquatic reptiles (two species of plesiosaur, one species of ichthyosaur, and the stem reptiles *Hovasaurus boulei* and *Barasaurus besairiei*). All size and shape measurements were collected in ImageJ (v. 1.53t). The long axis of each stone was measured manually using the “Straight Line” tool, and its circularity and roundness were computed in ImageJ by tracing the two-dimensional perimeter of each stone using the “Freehand selections” tool (see ImageJ User Guide: [71]). Annotated TIFFs containing all free-hand perimeter traces are available in our supporting information, and the associated measurements are given in S1 Table. We compared raw measurement distributions among bins with box-and-dot plots, and then Box-Cox–transformed all variables [72,73] to maximize their normality and homoscedasticity among groups prior to statistical analysis. We then performed a series of statistical tests, including one-way analyses of variance (ANOVAs) with post hoc Tukey tests to compare group means, and Levene’s tests with post hoc Tukey’s HSD on the ANOVA residuals to compare group variances. Statistical tests were performed using the lm(), aov(), and TukeyHSD() functions in the R *stats* package (v. 4.2.1: [74]; script S2), and the leveneTest() function in the package *car* (v. 3.1–2: [75]). Individual plots and outputs from statistical analyses were generated with the R package *ggplot2* (v. 3.4.2: [76]) and compiled into multi-panel figures with BioRender.com under the Student Plan Promo. All of the code used for statistical analysis and plot generation is available in our supporting information.

### Osteological and taphonomic characterization

Osteological and macro-petrographical analysis were performed under both natural and UV light in order to discriminate elements artificially re-attached with glue to the slab during restoration. Ultraviolet light was produced with a 95 W triple wavelength discharge lamp from WayTooCool LLC (UVA peak emission: 360 nm; UVB peak emission: 316 nm; UVC peak emission: 254 nm). Photographs were taken with either a Canon EOS 700D or with a Nikon D300S. 3D renditions of the slab’s elements such as isocurves and mapping of the depth differences were achieved by means of photogrammetry with Agisoft Photoscan. In order to test the composition of the gastroliths and adjacent matrix, six sediment samples were extracted from the slab for scanning electron microscopy–associated energy dispersive x-ray spectroscopy (SEM-EDS) analysis (S1: sample of matrix around G1; S2: sample of G2; S3: sample of G3; S4: sample of matrix around G5; S5: sample of greenish matrix near G6 and G9; S6: matrix around G6). SEM was carried out on a FEI Quanta 200 scanning electron microscope equipped with an EDAX Element-C2B detector (EDX), a backscattered electron detector and an Everhart–Thornley secondary electron detector housed at the CEASC (Centro di Analisi e Servizi per la Certificazione) of Padova University.

### Experimental acid etching

In order to compare the surface pitting of the *in situ* gastroliths with the surface etching caused by gastric acids, a set of eight unrelated pebbles (each around 2 cm long) of both carbonatic and siliceous composition (seven calcilutites and one agatha not from the Scaglia Rossa) were experimentally exposed to a synthetic gastric juice solution for different periods of time. A 500 ml solution was made with 5 ml of concentrated hydrochloric acid in 375 ml of milli-Q followed by the addition and dissolution of 2.5 g of dried pepsin and making up to the mark with milli-Q. Solution pH was corrected with NaOH 1 M up to 1.7, according to the acidity of extant tortoises reported by Wright et al. [77]. Pebbles were submerged in 15–20 ml of solution and kept in flasks at a constant temperature of 32°C in a bath of silicone oil. Pebbles were photographed before the treatment, after 48 hours and after 34 days. Solution in each flask was substituted every 2 days in order to mimic gastric secretions renewal.

### Gastrolith extraction from modern turtle gut contents

Gastrolith presence in extant sea turtles was investigated using from data collected from a previous study of green sea turtle (*Chelonia mydas*) diet in the Republic of Seychelles in the Western Indian Ocean (methods described in [33]). Samples from Stokes et al. [33] were re-analyzed to trace back the presence of gastroliths. As explained below, the frequency of gastroliths in the Seychelles samples was investigated between sexes and in gravid/non gravid females. Gravidity of females was confirmed by inspection of ovarian follicles > 2cm [78], and the majority of breeding females were captured on the nesting beach. A subsample (20–50%) of the original sample of gut content removed from the esophagus and upper cardiac region of the stomach biomass was analyzed for dietary content for the study by Stokes et al. [33]. Gastroliths were found in 71% of gravid female green sea turtles (n = 17) and < 5% of male or non-breeding females (n = 28). All gastroliths (> 5 mm) identified from remainder of gut content from four gravid females were carbonatic (n = 41). Dilute hydrochloric acid was used to test their carbonatic composition.

No animals were sacrificed specifically for this study. All sea turtle research was approved by Swansea University Ethics Committee (Ethics Reference Number: STU_BIOL_157334_011020182616_1; AWERB IP Reference Number: IP-2021-01). In the Seychelles, no turtles were killed specifically to provide diet samples for the study of Stokes et al. [33]. The necropsy was conducted under research permit (000XSE22) from the Commissioner’s Representative for the British Indian Ocean Territory (BIOT) and complied with all relevant local and national legislation.

## Results

### Systematic paleontology

TESTUDINES Linnaeus, 1758 [79]

CRYPTODIRA Cope, 1868 [80] *sensu* Evers et al. [81]

PAN-CHELONIOIDEA sensu Evers et al. [81]

PROTOSTEGIDAE Cope, 1873 [82]

cf. *Protosphargis veronensis* Capellini, 1884 [35]

Figs 2–4

**Fig 3:**
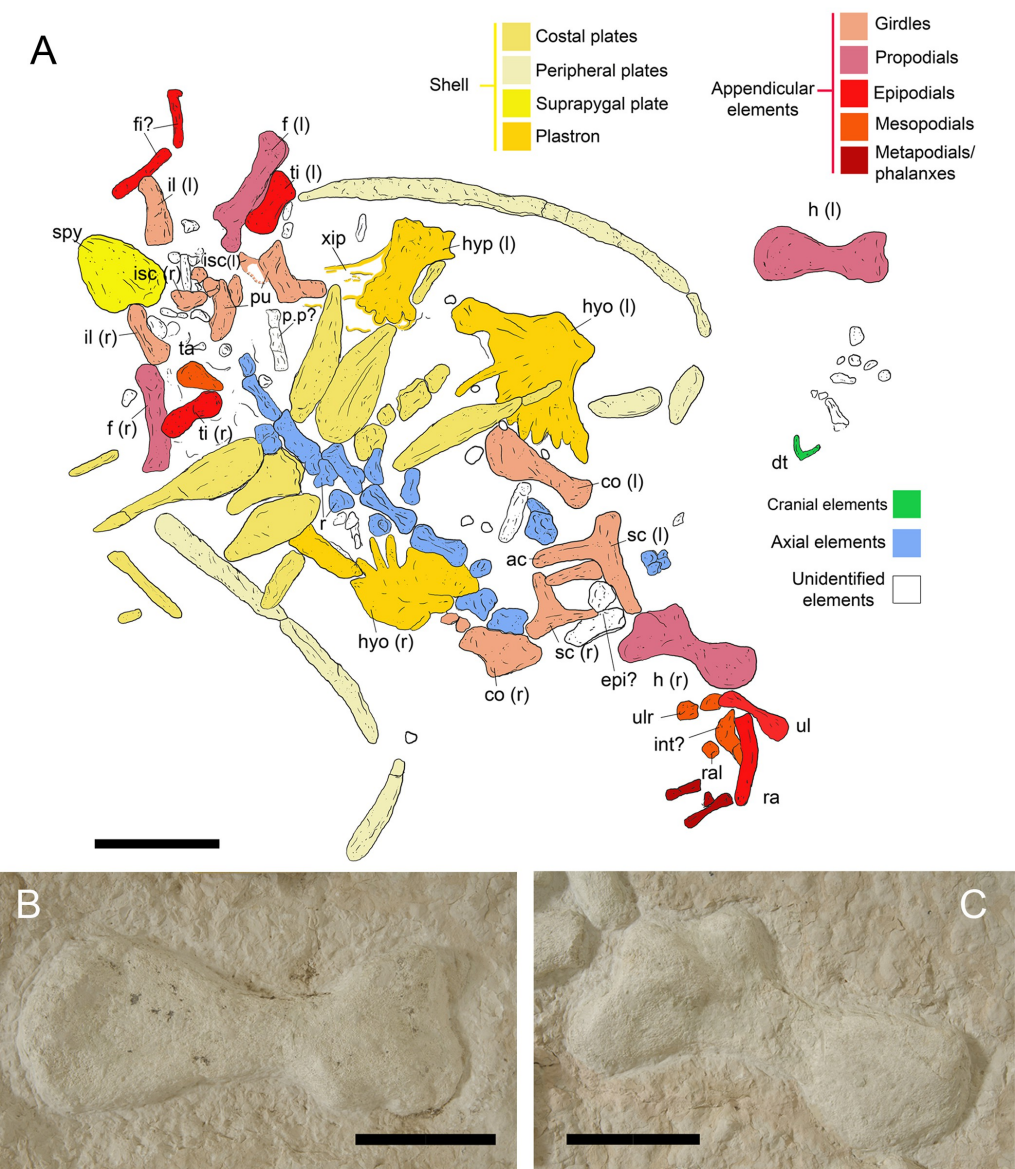
Anatomical interpretation of IGVR 91051. A) Anatomical drawing with color-based differentiation of skeletal regions. B) Detail of left humerus. C) Detail of right humerus. Abbreviations: ac, acromion; co, coracoid; dt, dentary; epi, epiplastron; f, femur; fi, fibula; h, humerus; hyo, hyoplastron; hyp, hypoplastron; il, ilium; int, intermedium; isc, ischium; p.p, peripheral plate; pu, pubis; r, rib head; ral, radiale; sc, scapula; spy, suprapygal; ta, tarsal; ti, tibia; ul, ulna; ulr, ulnare; xip, xiphiplastron. Scale bars: 10 cm.

**Fig 4:**
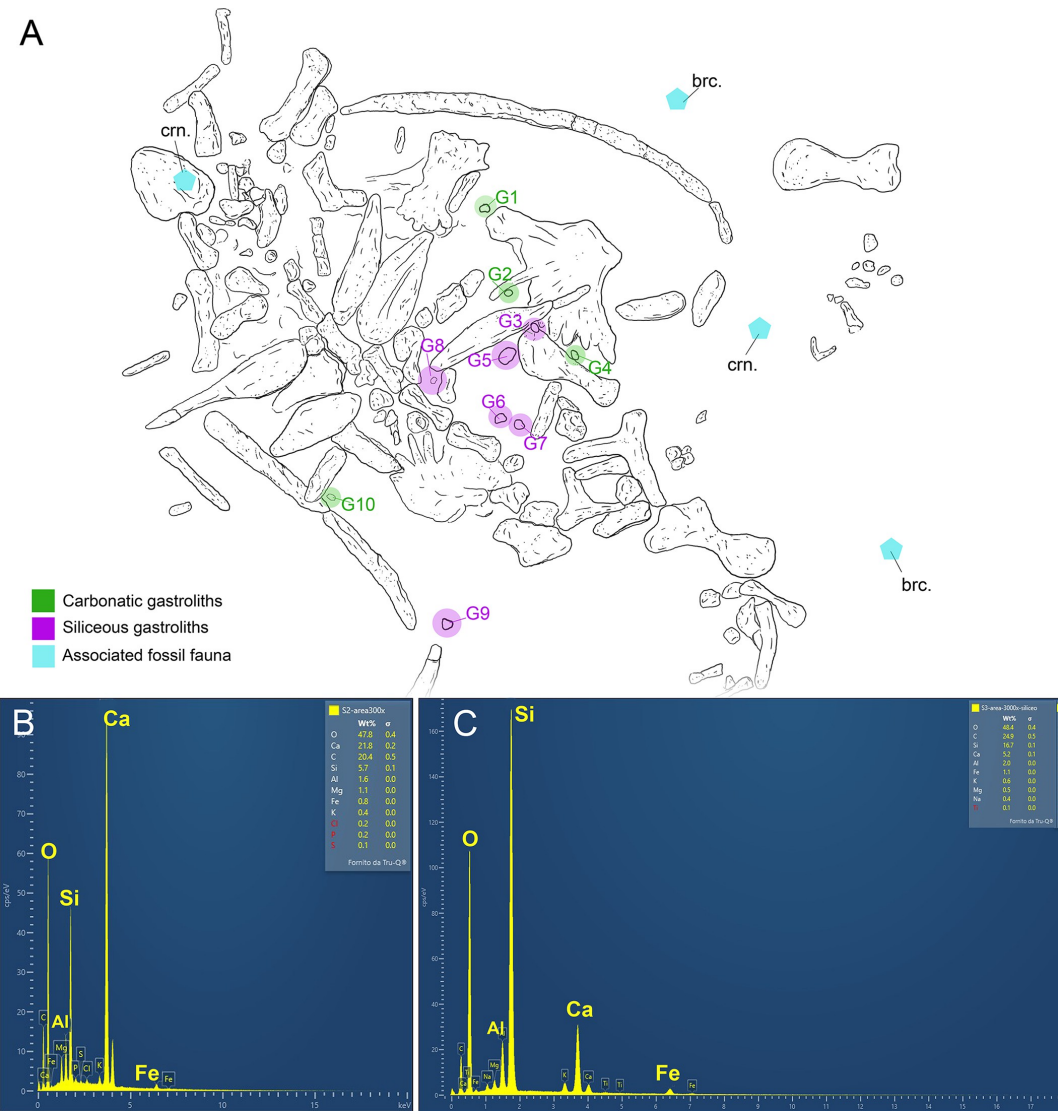
Gastrolith locations and compositions. A) Designation and distribution of the ten preserved gastroliths on IGVR 91051 and of the other materials foreign to the skeleton. B) SEM-EDS spectra of G3 highlighting its siliceous composition. C) SEM-EDS spectra of G2 highlighting its carbonatic composition. Abbreviations: brc., brachiopod; crn, crinoid element.

### Description

#### General features

The specimen IGVR 91051 lies exposed on the dorsal side (Fig 2). The specimen preserves almost exclusively postcranial remains, except for the anterior part of the dentary, which has a short symphysis. Several skeletal elements are partially articulated in anatomical connection, but the limbs and peripherals exhibit taphonomic loss (Figs 2 and 3A). Manus and pes are mostly lost. Beside the skeletal remains, the IGVR 91051 slab also preserves two brachiopods and crinoid stem elements (Fig 4A). Carbonatic and siliceous allochthonous pebbles are grouped anteriorly (Fig 4).

#### Shell

The shell is preserved as several associated elements of the carapace and the plastron. The carapace is strongly reduced, with large costo-peripheral fontanelles, which apparently extended more than half of the length of the ribs. There are only 4 pairs of costal elements preserved, with a general teardrop shape, expanded medially and tapering peripherally (Fig 3A). The teardrop shape of the costals exhibits some minor variations of lateral extension: the anterior-most elements have a posterior flange, the medial elements have an anterior and a posterior flange, while the posterior-most costals have an anterior flange. This teardrop pattern seems present also in another specimen (MCSNV V 10670; [40]: Fig 13B). Posterior to the right costal plate series, 3 sub-rectangular elements of dubious identity are recognizable (?posterior peripherals; Fig 3A). The lateral peripherals are very narrow, simple, and elongated elements, still in connection on the left side and completely displaced on the right side. A subtriangular, posteriorly tapering element placed caudally to the pelvic bones is interpreted as a suprapygal, present as a single element indicated by its large size (Fig 3A). The plastron is also strongly reduced, with central plastral fontanelles and reduced contact between hyo- and hypoplastra: IGVR 91051 preserves the left and right hyoplastron, heavily ossified and strongly frilled with thin digitations (also defined as strong serrations: see [81,83]), and the right hypoplastron, also characterized by a jagged margin (Figs 2 and 3A). The strong serrations of hyo- and hypoplastra are present along the surfaces that face other bones, but serrations are absent along the margin of the central fontanelle and the lateral contact area of hyo- and hypoplastra. The right hypolastron articulates with a rod-like posteriorly elongated and narrow element, interpreted as the proximal portion of the xiphiplastron. The xiphiplastron has an elongate anterolateral process articulating along the posterolateral margin of the hypoplastron, resulting in an oblique suture, and the hypoplastron extends posteriorly along the anteromedial margin of the xiphiplastron (Fig 3A).

#### Vertebral column

There are four cervical vertebrae, isolated in proximity to the pectoral girdle elements, and six trunk vertebrae, still in anatomical connection to each other, to rib heads and costal elements of the carapace (Fig 3A). The cervical series is represented by the axis and three procoelous posterior centra and disarticulated neural arches. The transverse process of the centrum is situated anteriorly and all centra are rounded in cross section as in protostegids and unlike in cheloniids and dermochelyids [81]; the posterior centra are greatly flattened. The trunk centra are elongated anteroposteriorly and narrow and articulate bilaterally with ribs heads. Rib heads are aligned with the junctions of adjacent vertebral centra.

#### Pectoral girdle

Elements of the left pectoral girdle (coracoid, scapula) are present. Scapula and coracoid are unfused at the level of the glenoid (Fig 3A). The right pectoral girdle is represented by the right scapula (with scapular and acromion processes visible and damaged) and a fragmented coracoid. The right scapula appears smaller than the left one, most likely due to diagenetic alteration or preparation damage. The internal angle of the acromion and scapular processes is ca. 115° (>110°). The acromion process is roughly as long as the scapular process. The coracoid is flat and narrow, approximately as long as the humerus and has a rather expanded asymmetric distal end.

#### Pelvic girdle

Several elements of the pelvis are preserved in IGVR 91051 (Fig 3A). Each pair of pubes, ischia and ilia are preserved but slightly scattered, but were possibly joined by cartilage when alive, as in young cheloniids and *Dermochelys coriacea* [84,85]. The pubis is a L-shaped flat bone with an expanded medial process in correspondence of the pubic symphysis and a lateral protruding process. The lateral pubic process is flat, square-shaped, and prominent: it extends anterolaterally, being deflected about 59° from the sagittal plane of the pubic symphysis. The lateral process protrudes anteriorly beyond the medial process. The medial process has a limited posterior extension. The ischium is broad and slightly triangular. The ischiatic symphysis is short, indicating a single large thyroid fenestra. The metischial process is not visible. The ilium is comma-shaped, rather broad and posteriorly elongated.

#### Forelimb

The left forelimb is represented only by the humerus and some purported distal carpal elements near the dentary. The right forelimb exhibits a partial articulation of humerus, ulna, radius and wrist elements (ulnare, intermedium, radiale) and three of the first phalanges. The right humerus is exposed on the dorsal side (Fig 3B) while the left one is exposed on the ventral side (Fig 3C). The humerus is longer than the femur. It has a slender shaft and an enlarged medial process, almost at the same level of the caput humeri; the lateral process is slightly squared, located distally to the caput humeri but along the proximal end of the shaft. The lateral process is also seemingly not restricted to the anterior surface of the shaft, but slightly expanded onto the ventral surface with a gentle protuberance (Fig 3B and 3C). The medial concavity of the lateral process is poorly preserved but apparently present. The distal articulation exhibits a rounded epiphyseal surface without clearly defined articulation facets. The radius is curved, approximately at mid-length. The radius length corresponds to ca. 68% of the humerus length. The ulna has the distal end slightly larger than the proximal end. The ulna is just over half the length of the humerus (ca. 58%).

#### Hindlimb

The hindlimb elements preserved in IGVR 91051 include both femora, the tibiae and three distal phalanges (Fig 3A). The femora are preserved *in situ* posterior to the tibiae, indicating taphonomic displacement. The femur is shorter than the humerus (ca. 77%). The shaft is straight and slender, the head is spherical, and the trochanters are well-separated from each other, without a connecting ridge between them. The tibial articulation is large and bulbous. The tibia is slightly more than half the length of the femur (ca. 63%), is stout and its shaft slightly broader than the femur shaft.

### Taphonomy of the specimen

**Fig 5:**
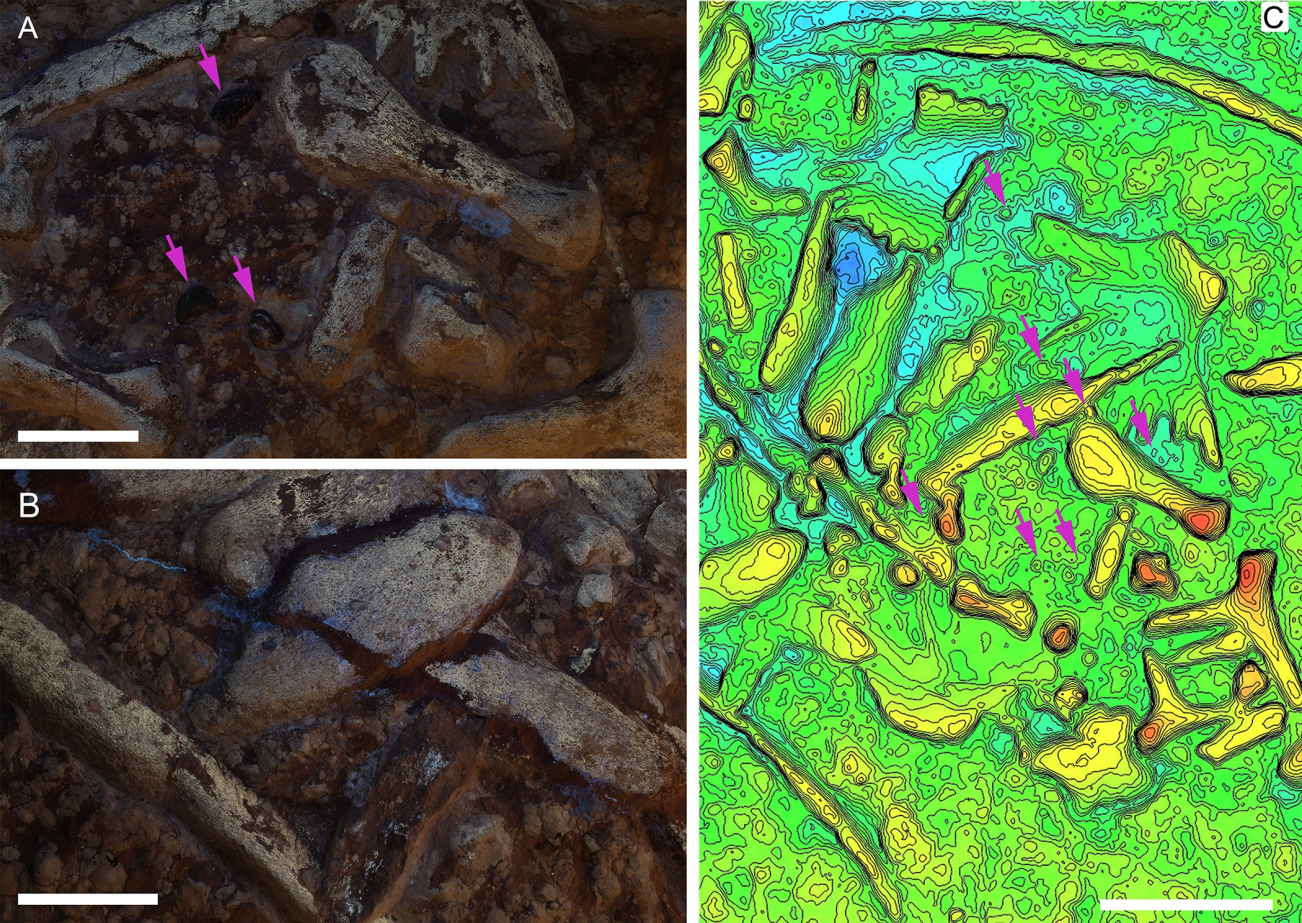
Preservational and taphonomic details of IGVR 91051. A) Anterior left region of the skeleton under UVA-B-C light showcasing the lack of fluorescence-response of the glue near the gastroliths. B) Detail of costal and peripheral carapace elements under ultraviolet light. C) Photogrammetry 3D depth map of the anterior portion of the specimen. Gastroliths indicated with magenta arrows. Scale bars: A) 5 cm; B) 10 cm; C) 30 cm.

IGVR 91051 exhibits a by-element completeness of 65–70%, with most of the carapace/plastron and axial skeleton still preserved, together with all four propodials and related girdles (Figs 1 and 2). The specimen is less complete distally towards the limbs. It also lacks the entire skull aside from a single dentary. Likewise, elements at the core of the skeleton (e.g. thoracic vertebrae, rib heads, costal plates and plastron components) are preserved in closer association, while they become disarticulated as one moves progressively towards the outside of the carapace (peripheral plates). Outside of the carapace area, each skeletal element seems instead to have drifted northeast (*sensu* slab orientation), most notably the right humerus. Peripheral elements do not appear to share a preferential orientation, excluding a possible sorting by bottom currents. Gastroliths are mostly grouped on the left side of the specimen, with only two being found on the animal’s right side (Fig 4A). Pebbles on the left side reside in a roughly 50X60 cm area, with some of the elements parallel one to another. The position of the gastroliths in relation to the original layout of the gut are later discussed in detail (see stomach necrolysis paragraph). UV analysis confirmed that the allochthonous clasts are genuinely attached to the slab and not glued, as artificially attached elements are clearly recognizable by the pale blue halo of the fluorescent glue/cement (Fig 5A and 5B). 3D photogrammetric maps highlight that gastroliths are less elevated than surrounding pectoral elements (Fig 5C), while they share similar depths of the plastron elements; this supports a ventral landing of the carcass on the seafloor, as the more ventral elements (stomach contents and plastron elements) reached the sediment first. The associated fossil fauna is too scanty to properly attribute its presence to a precise deadfall ecological stage of the carcass decomposition: future in-depth taphonomic studies on the fossil assemblage of all SAA tetrapods must be conducted before framing the role of brachiopods found around carcasses. Finally, the specimen skeletal tissue is mostly represented by exposed cancellous bone, suggesting chemical erosion of the compact bone by means of water dissolution. When preserved, the compact tissue presents sub-circular areas of depression, where the laminar cortical structure appears to have internally collapsed. This pattern, almost exclusively visible under UV light (Fig 5A and 5B), could also be consistent with bioerosion. Both chemical and biological erosion are an index of long exposure of the carcass on the seafloor before burial, which is consistent with the slow sedimentation rates estimated for the SAA [41].

### IGVR 91051 gastrolith characterization

**Fig 6:**
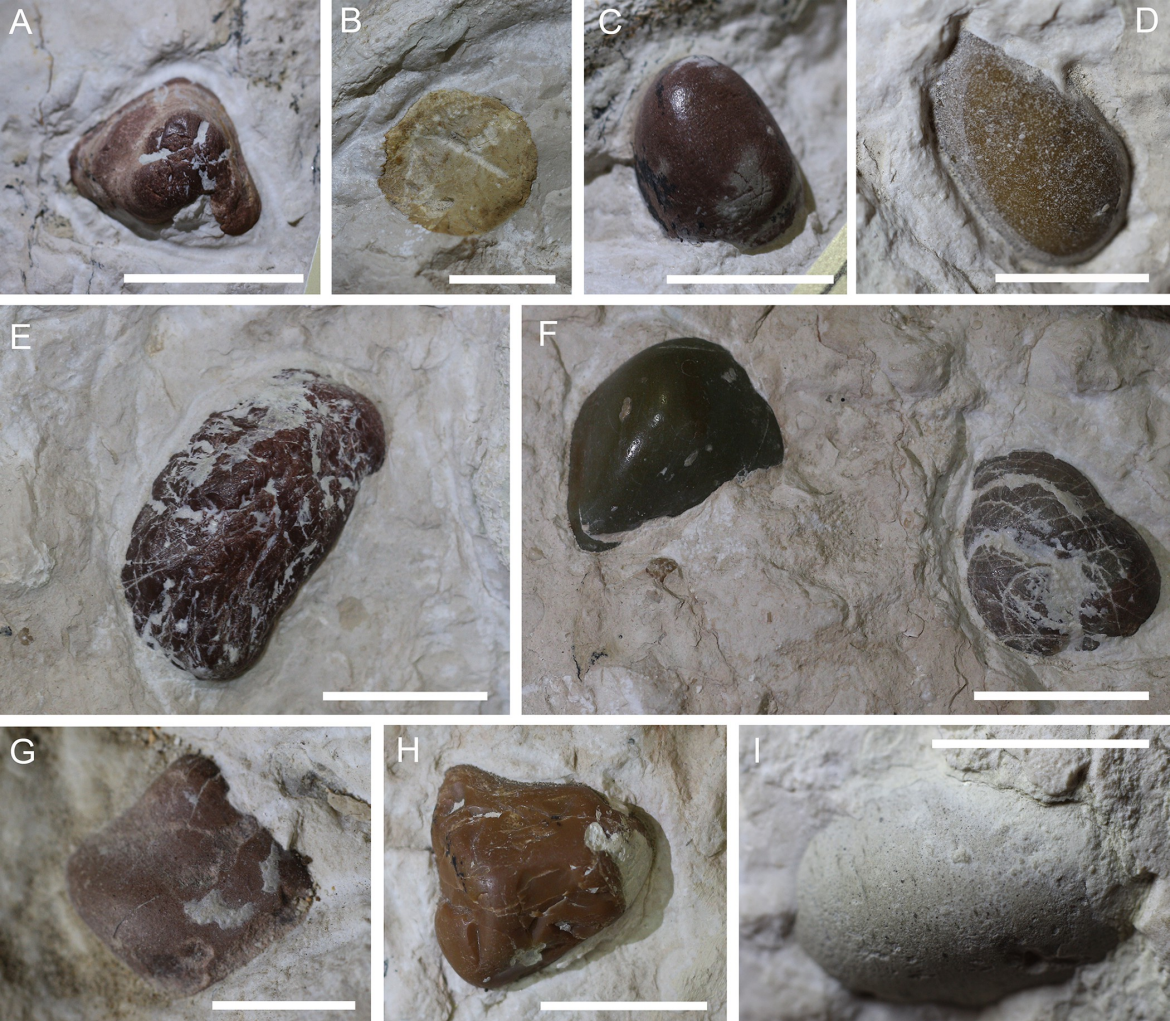
Close-ups of the preserved gastroliths within IGVR 9105. A) G1. B) G2. C) G3. D) G4. E) G5. F) From the left G6 and G7. G) G8. H) G9. I) G10. Scale bars: A,C,E,F,H 2) cm; B,G,I) 1 cm.

Ten rounded pebbles are exposed from the matrix between the axial and dermoskeletal elements of IGVR 91051 (Figs 4A and 6); these clasts are petrologically distinct from the surrounding sediment, as they differ greatly in shape and color from the pelagic limestone nodules of the slab. Preserved pebbles were labeled as G1 to G10, arbitrarily starting from the top right of the slab (Fig 4A). Eight pebbles are located on the left side of the specimen, while only two (G9 and G10) lay to the right of the vertebral column. A detailed description of each pebble, together with SEM-EDS data on their composition is reported in Table 1.

**Table 1:** Summary of gastroliths characterization in IGVR 91051.

	Size*	Roundness	Sphericity	Luster	Surface	Color	Composition	Figure
G1	2.4 x 1.9 cm	Sub-rounded	Low	Dull	Cracked and crossed by calcite veins, pitted	Rust-reddish	Carbonatic	6A
G2	1.9 cm Ø	Well-rounded	High	n.a.	n.a. (sectioned)	Light gold	Carbonatic	6B
G3	2.2 x 1.6 cm	Well-rounded	High	Vitreous	Mostly smooth	Dark red with gray patches	Siliceous	6C
G4	2.5 x 1.4 cm	Well-rounded	Medium	Vitreous	Densely pitted	Light brown	Carbonatic	6D
G5	5.3 x 2.4 cm	Sub-rounded	Medium	Vitreous	Rugose texture, crossed by cracks and scratches	Dark red	Siliceous	6E
G6	3 x 2 cm	Rounded	Medium	Vitreous	Smooth and glossy	Dark green	Siliceous	6F
G7	2.5 x 2.2 cm	Rounded	High	Dull	Roughened texture with cracks and tears	Dark brown	Siliceous	6F
G8	1.2 x 1 cm	Sub-rounded	Low	Dull	Smooth	Light brown-rust	Siliceous	6G
G9	2.8 x 2.6 cm	Sub-rounded	Low	Resinous	Smooth and glossy with cracks	Light brown-reddish	Siliceous	6H
G10	1.9 x 1.1 cm	Well-rounded	Medium	Dull	Densely pitted	Light gray	Carbonatic	6I

* Maximum outcropping width and height.

Only two of the preserved pebbles were directly sampled for SEM-EDS analysis (G2 and G3; Fig 4B and 4C), as the other clasts were too hard to be drilled with the available Dremel. However, additional samples were taken of the matrix peripheral to some pebbles to test if some small fragments had detached from the stones during acid etching. Samples of sediment adjacent to G1, G5 and G6 were sampled and revealed EDS spectra typical of a calcium carbonate substrate (with high peaks of calcium, oxygen, and carbon), consistent with the lithological composition of the slab. Widespread calcispheres, possibly attributable to dinoflagellate cysts, can be observed in these samples, ranging from globular to ellipsoidal in shape and approximately 50 μm in diameter.

### 2D morphometric comparison

**Fig 7:**
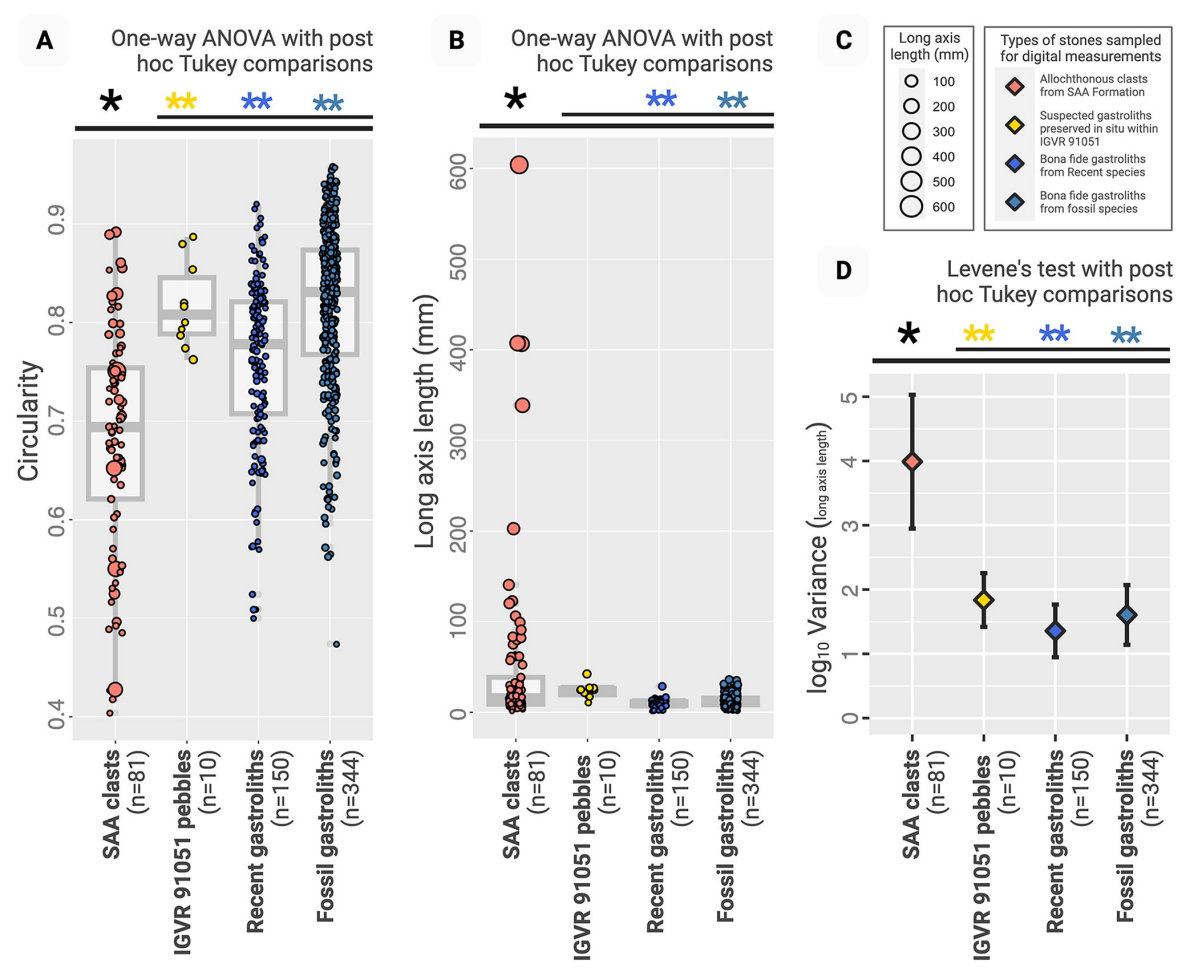
2D Morphometric analysis. A) Stones preserved within the trunk of chelonioid IGVR 19501 were significantly more circular than abiotically dispersed allochthonous clasts from the same formation (SAA), as were *bona fide* gastroliths from other species. B-D) *Bona fide* gastroliths were also significantly smaller than abiotically dispersed SAA clasts, which had significantly higher variance in size than both the stones preserved within IGVR 19501 and the *bona fide* gastroliths. Single asterisks (*) indicate significant among-group test results (ANOVA and Levene’s test *p* < 0.05), whereas double asterisks (**) indicate that the designated group differs significantly from the set of SAA clasts (pairwise post hoc Tukey tests, *p* < 0.05).

The set of suspected gastroliths within IGVR 91051 (G1–G10), along with the sets of *bona fide* fossil and Recent gastroliths (see S1 Table), were each significantly more circular than the SAA allochthonous clasts (Fig 7A; ANOVAs with post hoc Tukey tests: each *p* < 0.0001). In contrast, the IGVR 19501 stones (G1–G10) had circularities statistically indistinguishable from those of fossil and Recent gastroliths (post hoc Tukey tests: each *p* > 0.05). The suspected IGVR 19501 gastroliths thus bear a closer geometric resemblance to confirmed gastroliths (from varied taxa and preservational settings) than they do to abiotically dispersed clasts from their formation of origin. Surprisingly, roundness did not differ significantly between any two groups in our data set (ANOVA: *p* = 0.03; post hoc Tukey tests: each *p* > 0.05), indicating that the observed differences in circularity among groups did not reflect changes in the rounding of the individual corners of particles. Long axis length was significantly more variable in the SAA allochthonous clasts than it was for IGVR 91051 stones or the *bona fide* gastrolith groups (Fig 7B and 7C; Levene’s test with post hoc Tukey comparisons: each *p* < 0.05). All ten stones within IGVR 91051 fall within the size range of genuine gastroliths from our data set, whereas the abiotically dispersed SAA clasts span a far wider size range. Thus, in both shape and size, the suspected gastroliths within IGVR 91051 (G1–G10) resemble *bona fide* gastroliths far more than other studied allochthonous clasts from the SAA Formation.

### Experimentally etched pebbles

**Fig 8:**
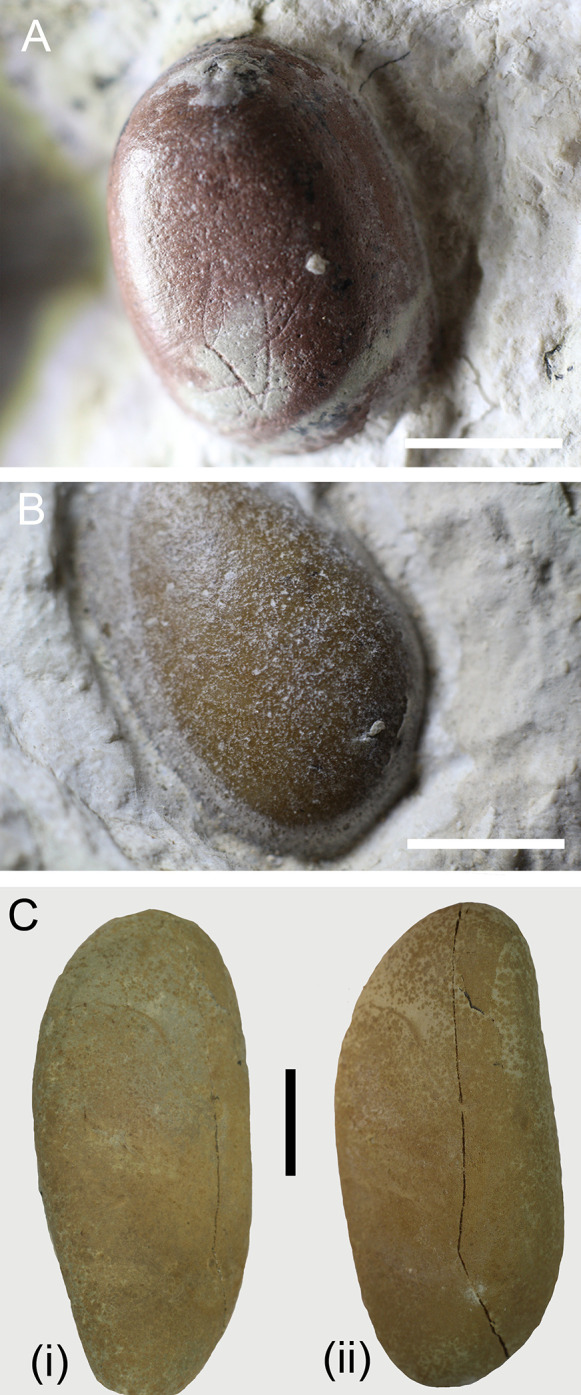
Surface etching patterns on carbonatic pebbles. A) Surface pits and cracks on G3. B) Surface pitting of G4. C) Experimentally etched pebble before (i) and after a month (ii) of treatment. Scale bars 5 mm.

The seven carbonatic pebbles used to extrapolate surface etching patterns in experimental settings displayed visible changes in their surface texture after exposure to HCl and pepsin: pre-existing scratches and crevices are highlighted after the 48h treatment, but are deepened after the 34 days. Most notably, pitting of the surface is drastically increased after the full treatment; the fifth sample (C5) is illustrated in Fig 8 in comparison to IGVR 91051 gastroliths. Pitting in C5 is most consistent with the surface pattern found on G4 and G10. Changes in roundness and smoothness were not detected in the experimentally etched pebbles, most likely due to the absence of tumbling action. However, almost every carbonatic pebble experienced a loss of 0.1 g (between 3.8 and 12% weight loss) by means of dissolution after the 36-day treatment. Siliceous pebbles did not experience any appreciable change after the full treatment, which is consistent with previous studies (e.g., [2]) and the absence of distinct surface pitting in the siliceous gastroliths of IGVR 91051.

### Geo-gastroliths characterized in modern turtles

**Fig 9:**
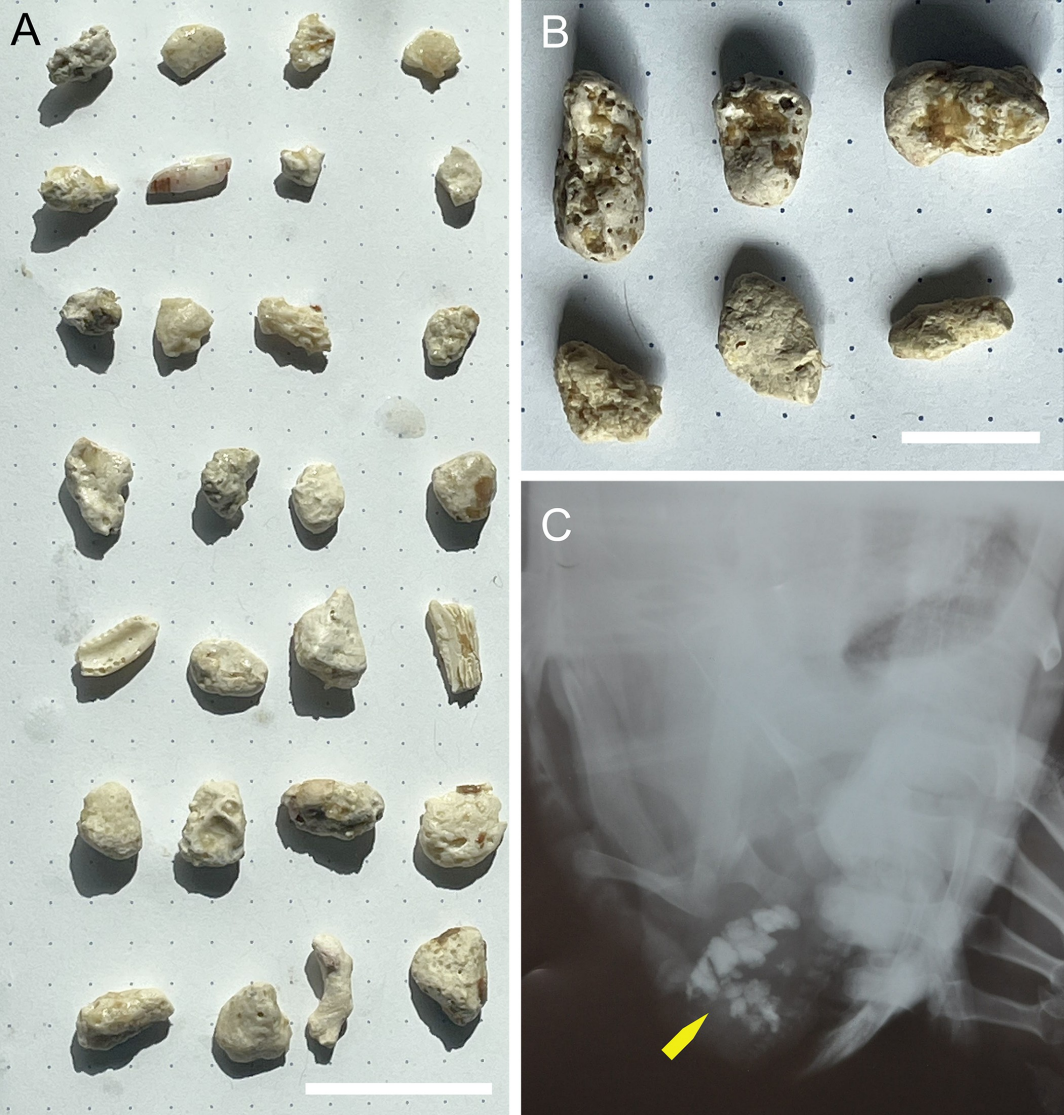
Extant chelonioid gastroliths. A) 28 carbonatic elements extracted from the gut contents of *Chelonia mydas* sample #23 retrieved from the Republic of the Seychelles. B) 6 carbonatic elements extracted from the gut contents of *Chelonia mydas* sample #10 retrieved from the Republic of the Seychelles. C) Radiograph of a 20-year-old female *Eretmochelys imbricata* from the Red Sea with gravel in the colon (image courtesy of Efrat Weizman Kohavi, Israeli Sea Turtle Rescue Center). Scale bars: A,B) 2 cm.

In extant green sea turtles examined for this study, gastroliths were found with a frequency of 71% in gravid females (n = 17) and < 5% of males and non-breeding females (n = 28) sampled in the Western Indian Ocean (Table 2). In the four gravid females from which stones were extracted, all identified gastroliths were carbonatic in nature (n = 41). Retrieved ingested clasts were mostly composed of coral fragments (Fig 9A and 9B), consistent with the availability of carbonate sediment particles in the Seychelles coral atoll. In one gravid female (sample 23, S1 Table), bivalve shell fragments were also identified among gastroliths. Gastroliths from the Seychelles dataset possess uniformly pitted surfaces, and, although rounded, they appear variable in shape, most likely due to the action of gastric acid on the “softer” aragonitic material. No siliceous particles were identified in any of the specimens sampled. We also contacted several marine reptile rehabilitation centers for information on gastrolith-bearing turtles, and present here applicable results with their permission. In particular, we showcase a radiograph of a 20-year-old female *Eretmochelys imbricata* from the Red Sea provided by the Israeli Sea Turtle Rescue Center that contains gravel in its colon (Fig 9C); this specimen highlights how ingested particles eventually transit to the end of the digestive system.

**Table 2:** Gastrolith distribution in the surveyed Seychelles dataset. Gastroliths (coralline rock fragments) were found in the majority of guts from gravid female green sea turtles (*Chelonia mydas*) but not in non-breeding female or male individuals in a study of green turtles legally harvested in the Republic of Seychelles in 1982–1983.

	Male	Non-breeding female	Gravid female
Number of individuals (n)	26	2	17
Relative proportion	*	*	15±6
FO (%)	4	0	71

Data represent relative proportion of gastroliths in the biomass of dietary components (mean ± SE) and frequency of occurrence (FO) of gastroliths. Further details provided in [33].

*<0.1mg (trace)

## Discussion

### Taxonomic discussion

The phylogenetic relationships of fossil marine turtles are strongly debated (e.g., [81,83,86,87]). Among fossil marine turtles, protostegids have been recovered as stem-cryptodirans (e.g., [88–94]), stem-chelonioids [81,86], or crown-chelonioids as the sister group to dermochelyids [83,95–98]. While a precise taxonomic placement is pending a full phylogenetic analysis, IGVR 91051 exhibits a suite of characters that tentatively allow us to attribute this specimen to the total-group Chelonioidea *sensu* Evers et al. [81]: reduced carapace and plastron with presence of costo-peripheral and central plastral fontanelles; serrations on the margins of hyo- and hypoplastra; the position of the lateral process of the humerus, located distally to the caput humeri but along the proximal end of the shaft; the humerus length greater than the femur length. The specimen also exhibits a few other characteristics that are considered synapomorphic features of crown-group Chelonioidea and shared by Protostegidae: absence of cervical scutes; xiphiplastral shape as anteroposterior elongate rods; internal angle between the acromion and scapular processes greater than or equal to 110°; coracoid length similar to humerus length; and a rounded distal articular surface of the humerus without clearly defined articulation facets [81]. Furthermore, IGVR 91051 exhibits at least two characters which are autapomorphies of Protostegidae: radius with curved shaft [81]; lateral process of the humerus expanded onto the ventral surface of the shaft [83]. Moreover, the specimen seems to possess a single suprapygal as shared by Protostegidae [81] though additional elements might have not been preserved. A relatively high taxonomic diversity of marine turtles from the “lastame” has been reported [45,64,99]; however, the largest specimens so far recovered are referred to the taxon *Protosphargis veronensis* (e.g., [37,43,45). IGVR 91051 exhibits many similarities in the shell and humerus morphology with the holotype of *P*. *veronensis* and other material provisionally referred to this taxon ([35,37,40,43,45,61]). For this reason, the specimen described herein is provisionally identified as cf. *P*. *veronensis*. The taxon *Protosphargis* has been referred to Protostegidae (e.g., [95,96,100]). However, the necessary thorough morphological revision to investigate its phylogenetic affinities is beyond the scope of this paper. The characters herein described nonetheless support the provisional referral of the taxon to Protostegidae, pending a comprehensive taxonomic revision of the marine turtles from the “lastame”.

### The pebbles preserved within IGVR 91051 are geo-gastroliths

We present different lines of evidence that strongly support the gastrolith nature of the pebbles preserved inside IGVR 91051. Firstly, these allochthonous stones have not been artificially attached on the slab during restoration as highlighted by UV-light fluorescence (Fig 5A), and they are localized exclusively inside the thoracic area of the specimen (Fig 4A). In addition, they have sizes and shapes more akin to those of bona fide geo-gastroliths from other fossil and extant taxa than abiotically dispersed dropstones naturally occurring in the SAA (Fig 7). SEM-EDS spectra, coupled with macroscopic petrographic identification, highlight a compositional variability in the preserved pebbles, which contrast with the compositional homogeneity reported for authigenic mineral precipitates that can be mistaken for gastroliths (e.g. [3]). Moreover, the pattern of surface pitting on the carbonatic pebbles in IGVR 91051 is comparable with that of experimentally etched calcilutites treated with HCl and pepsin to mimic turtle gastric solution (Fig 8C). All lines of evidence thus support that the pebbles preserved within this specimen are gastroliths. Since these gastroliths are structurally and chemically recognizable as naturally occurring sediments rather than pathological calcium oxalate concretions, they can be described as geo-gastroliths, external sediment particles ingested by the animal. IGVR 91051 therefore represents the first reported case of a fossil turtle hosting geo-gastroliths.

### Implications for necrolysis

Assuming the surface of the slab to represent the upper bedding plane of the stratum, the specimen landed ventrally to the seafloor; despite the dermatoskeleton being greatly reduced in protostegids, the carapace appears to have shielded the core elements from scattering (e.g. pelvic girdle). The ten gastroliths in IGVR 91051 are mostly grouped in close proximity, suggesting also little scattering during necrolysis of the digestive tract. G1 to G8 are found in an arc on the left side of the specimen’s main body axis (Figs 2 and 4A), which is consistent with the position of the “J” shaped stomach in turtles and most sauropsids [101–103]. Interestingly, at least two subgroups of pebbles on the left side are found arrayed in two parallel lines (G8, G6, G7 and G3, G4; Figs 4A and S1). The peculiar arrangement of these pebbles could be coincidental, as the action of currents might be responsible for the movement of these rounded elements on a muddy seafloor, while the surrounding pectoral girdle bones could have influenced the final position of the gastroliths (i.e. G3 and G4 resting between left coracoid and hyoplastron). An alternative hypothesis might rely on an anatomical explanation. The stomachs of most extant turtles present a gastric relief with regularly spaced, robust and smooth longitudinal folds parallel one to another [101,102,104] (S1 Fig). These folds have different patterns of density and pronunciation throughout the stomach compartments of living Chelonioidea [103,105]. Longitudinal folds of mucosa are straight but can become more convoluted towards the cardia and pylorus, persisting to the duodenum [102]. A possible interpretation of the peculiar two-lined arrangement of gastroliths in IGVR 91051 is that they might have resided between, or inside, longitudinal folds, either from the stomach or duodenum. In this scenario, the pebbles might have helped to pin down the stomach musculature during necrolytic detachment from the esophagus and small intestine, which then resulted in the preservation of some remnants of the gut content not far from the anatomical position. We are aware that this speculative explanation requires more solid observations on additional specimens. Thus, at present, whether the arrangement of pebbles within the digestive tract reflect its soft-tissue anatomy or random taphonomic processes remains unclear.

### Gastroliths in Chelonioidea

Even in extant vertebrates, geophagy is a controversial topic, and its function in marine tetrapods is often not fully understood. One of the most historically popular theories regarding the consumption of gastroliths by marine reptiles and marine mammals is that they might provide ballast and buoyancy control [8,16]. Although it has received some recent support in alligators [24], this theory was challenged by Everhart [29], Henderson [106] and Wings [1], who suggested that gastrolith weights should have minimal influence on the buoyancy or stability of aquatic megafauna (e.g. plesiosaurs; [106]), including sea turtles ([8]). We would tend to exclude the possibility that pebbles in IGVR 91051 or in the Seychelles and Red Sea specimens had a ballast or hydrostatic-control function, since their cumulative weight (a few hundred grams) is negligible compared to the turtle mass, and since their presence seems sex-specific. Pathological explanations of geophagy are also considered controversial: disease- or stress-induced ingestion of substrate has been observed in modern tetrapods (especially in captivity), but likely represent isolated events [1]. Similarly, the deliberate ingestion of sediments to eliminate parasites (eg, intestinal helminths), although reported in scientific literature (e.g. in cormorants; [17]) is a behavior difficult to generalize in different groups of geophagic vertebrates. In contrast, alimentary hypotheses are generally regarded as more plausible explanations for geophagy [1], with direct observational lines of evidence supporting the use of gastroliths for grinding and mixing ingesta in various groups of extant vertebrates, together with the consumption of substrate as a mineral supplement. For extant turtles, it has long been hypothesized that breeding females ingest calcareous material to provide calcium for eggshell production, restore depleted calcium reserves or neutralize stomach acid whilst fasting [32]. These factors are important for extant green sea turtles that may lay a mean of six egg clutches containing > 120 eggs at two-week intervals during a nesting season, as eggs of each clutch are shelled before oviposition [107,108]. Here we suggest that gastroliths are important dietary components for extant breeding female green turtles in the Republic of Seychelles, as evidenced by presence of calcareous gastroliths in the intestinal tract in the majority of gravid females but rarely present in non-gravid females or male green sea turtles from the same location (Fig 8A and 8B; Table 2). Similarly, calcareous substrates were identified in stomach contents of gravid green sea turtles nesting on Caribbean beaches of Costa Rica and Raine Island, Australia [32,109], and gravid hawksbill turtles (*Eretmochelys imbricata*) in the Caribbean [110]. In contrast, stomach contents examined in male and non-breeding female green sea turtles foraging on seagrass in Nicaragua contained little or no calcareous matter [111]. Similarly, the ingestion of Ca-rich substrate was observed in terrestrial [10] and freshwater tortoises [112], leading the respective authors to discuss mineral supplement as a plausible explanation for geophagy. On the other hand, accidental ingestion of sediment was also reported in leatherback sea turtles [34] females and males alike, but such occurrences only involved non-selected and loose material (sand).

### Was IGVR 91051 a gravid female?

Taken in comparison with modern chelonioids, our discovery of gastroliths within the fossilized shell of IGVR 91051 provides evidence that the individual was potentially a gravid female marine turtle, which may have been close to a nesting beach or traveling to return to a foraging ground after nesting. This is supported by the nature of the gastroliths as terrigenous pebbles, most likely from a beach or estuarine setting; such environments tend to be visited by modern marine turtles only for nesting and egg deposition. The consumption of sediment particles could have very well functioned as a calcium supplement after (or in preparation for) egg production. Cadena et al. [113] demonstrated that Cretaceous protostegids developed egg shells that were much thicker than those of modern chelonioids, which would have made their calcium intake requirements particularly demanding. From this perspective, the ingestion of siliceous clasts together with the carbonatic ones should be considered coincidental, suggesting that protostegids had no chemico-physical way to distinguish calciferous and non-calciferous sediments. We should not, however, *a priori* exclude other possible explanations for the presence of gastroliths in IGVR 91051: the ingestion of pebbles could have been accidental or could have facilitated mechanical digestion of food being processed in the stomach. Kear [114] describes inoceramid bivalve fragments in the gut contents of *Notochelone* (c.f.) from the upper Albian of Queensland, suggesting that some protostegids, as already hypothesized by Hirayama (1994), fed on hard-shelled mollusks, a food class that could have benefited from trituration by gastroliths. We note, nevertheless, that the dentary on IGVR 91051 is thin and gracile, seemingly unsuited for a durophagous diet, which may suggest a more soft-bodied prey specialization for *Protosphargis veronensis*, in which case gastroliths would not have been needed as a digestive aid. Moreover, no mollusk shell fragments are found associated with gastroliths in IGVR 91051, and the associated brachiopods and crinoids are not related with gut content. While we do not exclude these alternative explanations, we think that our hypothesis better fits the available lines of evidence. The proposed function of ingested particles as a calcium supplement in gravid females could also explain the overall rarity of gastroliths in fossil marine turtles. If gastroliths had food-grinding functions (or other general alimentary functions) in Cretaceous chelonioids, their presence would have been more common in the fossil record. Our hypothesis, deeply rooted in the dietary habits and life history of modern sea turtles, justifies the rarity of gastroliths in fossil marine turtles, as it is likely that, in order to preserve ingested stones, the specimen had to be female, at sexual maturity, in breeding season and near egg deposition, besides also being fossilized in the right conditions.

## Conclusions

In this study we provide the first description of IGVR 91051, a large chelonioid from the Turonian of Sant’Anna d’Alfaedo, northeastern Italy. The specimen is the first fossil marine turtle to be reported with gastroliths preserved in its body cavity. Our study highlights the following results:

- IGVR 91051 can be ascribed to Protostegidae based on propodial and epipodial morphology; within Protostegidae, the specimen is tentatively ascribed to *Protosphargis veronensis* [35] due to humeral and shell anatomy.
- The taphonomy of the specimen suggests little disturbance on the seabed by currents and a long exposure before burial underlined by the strong erosion of the skeletal tissue.
- The specimen preserves 10 allochthonous pebbles (carbonatic and siliceous) between anterior plastron elements; 8 of these pebbles are grouped on the left side of the specimen, in a layout consistent with the position of the “J” shaped stomach of modern turtles.
- A 2D morphometric analysis highlights that these pebbles are size-selected, and statistically more similar in size and shape to gastroliths from other fossil and extant taxa than they are to abiotically dispersed dropstones from the SAA.
- The superficial pitted pattern of IGVR 91051 carbonatic gastroliths closely resembles the etching profile of carbonatic pebbles experimentally subjected to HCl and pepsin.
- The majority of the gastroliths are preserved on the left side of the animal’s axis. This is consistent with the anatomical position of the stomach of many sauropsids and suggests little scattering of the gut content during soft tissue decay.

Moreover, our investigation of geophagy in modern sea turtles revealed that:

- The majority of Seychelles specimens from which gastroliths were retrieved in gut contents were gravid females, suggesting a link between the consumption of substrate and reproductive physiology. This percentage is also consistent with other rare gastroliths-bearing specimens reported in the literature and veterinary observations.
- The consumption of carbonate clasts by marine turtles may serve as a behavioral means to supplement calcium intake during egg-shell development.

While we cannot exclude the possibility that IGVR 91051 consumed gastroliths either accidentally or as a digestive aid for the processing of ingesta, we propose that the studied specimen was likely a gravid female, thanks to modern analogues comparison. This study thus solidifies our understanding of geophagy in extant chelonioids and extends the history of this behavior back to the Cretaceous.

## Supporting information

S1 Fig Gastrolith arrangement on IGVR 91051. A,B) Lined gastroliths in both picture and anatomical drawing possibly matching former stomach foldings. C) Cropped stomach and partial small intestine of a female *Chelonia mydas* during necropsy (photo courtesy of Ellen Wood) with detail (D) of longitudinal folds in the mucosa highlighted in blue.
(TIF)

S1 Table Gastroliths and allochthonous Scaglia Rossa-“lastame” dropstones used in the 2D morphometric analysis. (XLSX)

S1 File R script for the 2D morphometric analysis in Fig 7. (TXT)
