# Supplementary figures and images for: First evidence of marine turtle gastroliths in a fossil specimen: Paleobiological implications in comparison to modern analogues
Source: PLoS One. 2024 May 6;19(5):e0302889. doi: 10.1371/journal.pone.0302889 (PMC11073738; doi:10.1371/journal.pone.0302889)

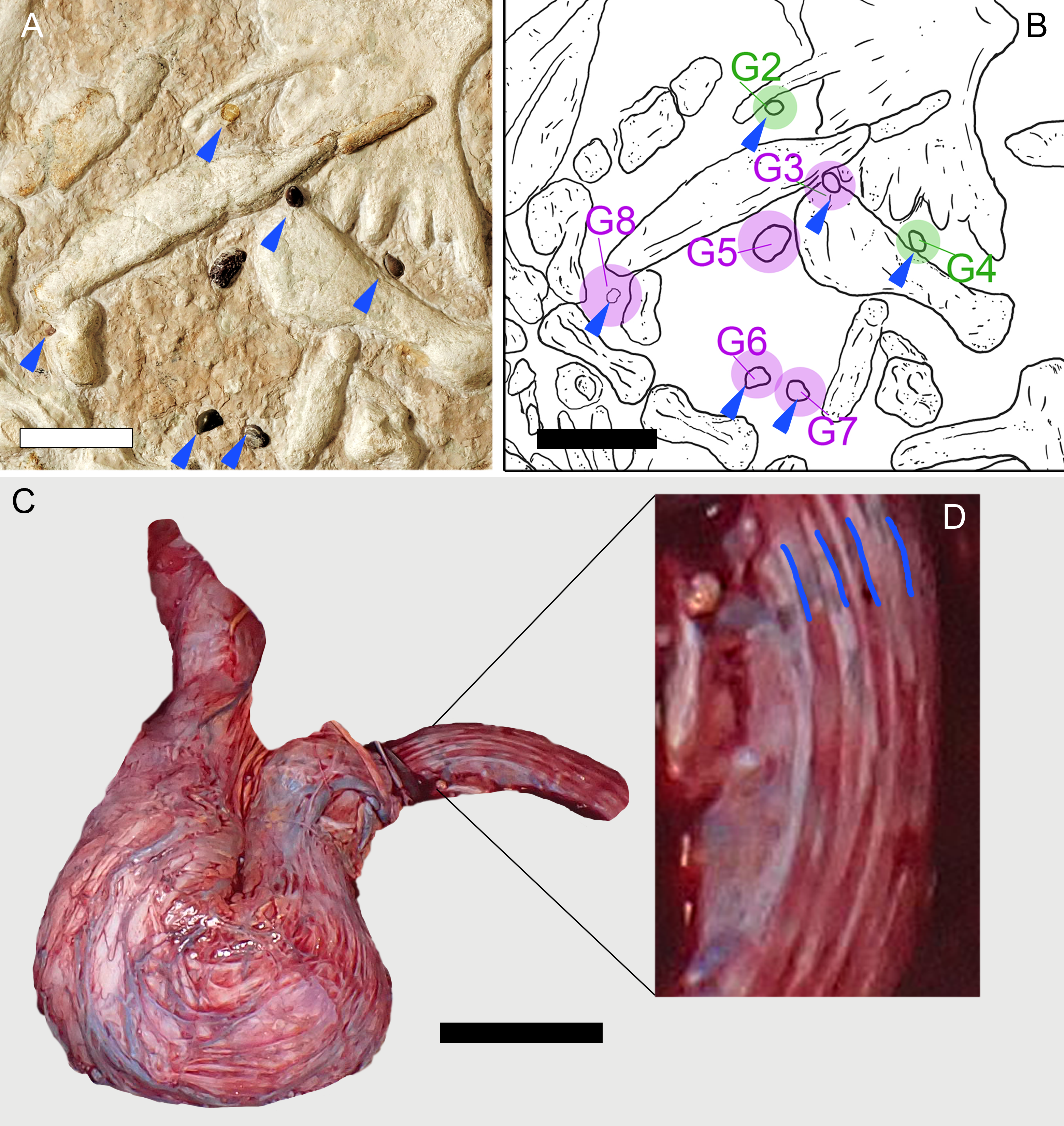

Supplement: S1 Fig — A,B) Lined gastroliths in both picture and anatomical drawing possibly matching former stomach foldings. C) Cropped stomach and partial small intestine of a female Chelonia mydas during necropsy (photo courtesy of Ellen Wood) with detail (D) of longitudinal folds in the mucosa highlighted in blue. (TIF) [file pone.0302889.s001.tif]
